# Promising Results of the Comparison of Coatings on Aged Bridges and of Same Coatings in Laboratory

**DOI:** 10.3390/ma15093064

**Published:** 2022-04-22

**Authors:** Agnieszka Królikowska, Leszek Komorowski, Ewa Langer, Małgorzata Zubielewicz

**Affiliations:** 1 Road and Bridge Research Institute, 03302 Warsaw, Poland; lkomorowski@ibdim.edu.pl; 2 Institute for Engineering of Polymer Materials and Dyes, 87100 Torun, Poland; ewa.langer@impib.lukasiewicz.gov.pl (E.L.); malgorzata.zubielewicz@impib.lukasiewicz.gov.pl (M.Z.)

**Keywords:** anti-corrosion coating, corrosion, coating, paint, anti-corrosive pigments

## Abstract

Many factors contribute to the high durability of anti-corrosion coatings. The most frequently mentioned are: appropriate protection design selected for the operating conditions, type of protection-type of metal and/or coatings, surface preparation, and proper application. Particular emphasis is placed on the type of protective materials. A lot of research is also carried out in this direction. In this article, we want to show that the standard protection with an epoxy/polyurethane system with thickness as recommended in ISO 12944-5: 2019, without special active fillers, is able to ensure high durability in a C4/C5 environment. This is confirmed by the presented results of electrochemical analysis, visual evaluation of coatings and adhesion of coatings and allows the use of well-known, inexpensive paint systems, assuming greater emphasis on their proper application. The results of the assessment of coating systems on bridges were used for comparison with the results obtained in various types of accelerated tests of the same coating systems and to make the selection of the optimal version of the laboratory tests.

## 1. Introduction

The effects of aging on standard anticorrosive coating systems (epoxy/polyurethane without zinc pigments) used to protect large infrastructure structures are rarely reported in European scientific journals as far as corrosion is concerned. It seems that this gap should be filled for several important reasons. First of all, this type of protecting coating accounts for approx. 80% of the total amount of systems, e.g., on bridges. In road and rail infrastructure (in heavy corrosion protection, epoxy coatings account for 40–60% of the global market [1]), it is an economic solution [2] as the average price of EP/PUR coating systems are cheaper and easier to apply in comparison to coatings with active anticorrosive pigments and flake fillers. Secondly, it is a solution available for use by standard experienced companies, and on the market, you can find systems suitable for all atmospheric corrosion hazards included in ISO 12944-2: 2017 [3] as well as systems suitable for use in assembled structures both in workshops and on-site, both in newly built objects and on old ones under maintenance work [4,5].

It is important that both designer and investor be convinced that it is a good answer ensuring long and very long durability (according to ISO 12944-1: 2017 [3]), if it is properly applied. This will allow for a conscious choice of durable and economical proposal and for the conscious refusing of more difficult/expensive challenges, often proposed by their distributors as the only answer meeting the requirement of high durability. Therefore, in this article, we would like to share our experience in assessing this type of protection on bridge structures in the C4/C5 corrosion hazard zones after approx. 15 years of operation.

At the same time, accelerated tests of the same systems used on bridge structures were carried out in the laboratory in order to select such tests that best simulate the actual damage on the structures after a long period of operation according to ISO 12944-1: 1998 [3]. This allowed for the selection of accelerated studies and their duration reflecting the degradation in the tested atmospheres. The optimum approach to making accelerated corrosion testing reliable is to compare its results with the results of long-term aging of coatings under natural conditions. This approach reduces the time required to evaluate the durability of coatings to the time of accelerated testing, which is many years.

The recommended accelerated tests were used in the requirements for anti-corrosion systems on bridge structures tested in order to obtain National Technical Assessments in accordance with the Regulation of the Minister of Infrastructure and Construction on the method of declaring the performance of construction products and the method of marking them with a construction mark of 17 November 2016 [6].

## 2. Materials and Methods

### 2.1. Bridge Structure Assessed

The tested coatings were used on the objects located in environments with corrosivity categories C4 and C5I in accordance with ISO 12944-2: 1998, after a service life of 10 to 17 years. Table 1 lists the tested objects and shows them in the photos. The systems used were made up of epoxy primer in different versions:

(1) Without active fillers;
(2) With zinc filler 75 and 94% by weight;
(3) In a dry shell and with an aluminum filler 2–4% by weight;
(4) Made of epoxy interlayer without active or barrier fillers;
(5) With aluminum filler 2–4% by weight and iron flake 12, 36 and 58 wt.%;
(6) With a polyurethane topcoat, predominantly acrylic;
(7) In one case acrylic/polyester.

Detailed data on the coatings used in the investigated paint systems are given in Table 2. All coatings are commercial products of large companies on the market and at the time of application had valid IBDiM (Road and Bridge Research Institute, Warsaw, Poland) Technical Approvals. The required tests for Technical Approvals can be found in [7].

#### 2.1.1. Coating Damage Assessment

On all bridge structures, the assessment of the condition of the coatings (corrosion, blistering, cracking, flaking, and chalking) was performed in accordance with the relevant part of ISO 4628-1 ÷ 5:2003 [8] and ISO 4628-6:2011 [8]; adhesion tests between layers to the substrate were carried out through the cross-cut method according to ISO 16276-2: 2007 [9], and thickness test according to ISO 19840: 2009 [10]. Coatings thickness was determined using Elcometer 456 electromagnetic measuring device (Elcometer, Manchester, UK). The instrument indications were verified with thickness standards RJ02783 (23.6 µm) and RF32126 (495 µm) as the average from 100 measurements on each area studied.

#### 2.1.2. EIS Studies of Coatings on Objects

**Figure 1:**
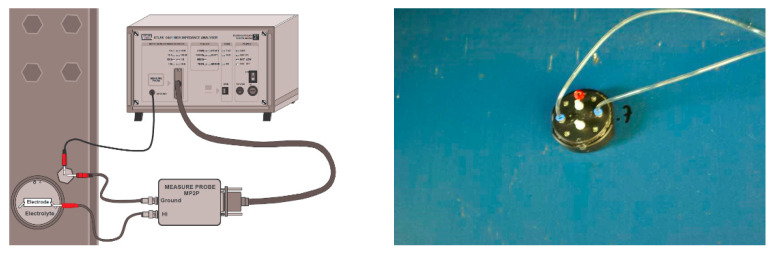
Set-up of the EIS measurement device.

In selected places with different corrosive exposures, the coatings were tested by electrochemical impedance spectroscopy in order to determine their barrier properties and to check whether there were undercoating corrosion processes. This type of research has been performed on bridge structures by the authors since 2000 [11], and their use has become a valuable and widely used tool for assessing coatings and the degree of advancement of underpaint corrosion processes [12,13,14,15]. The measurements were carried out using the Atlas analyzer (ATLAS 0441 High Impedance Analyser, Atlas-Sollich, Gdansk, Poland) in a two-electrode system, in a 3.5% sodium chloride solution, within a frequency range of 10^5^ Hz to 10^−2^ Hz. The working electrode was the sample itself and the counter electrode was a platinum mesh. The connection diagram is shown in Figure 1. The impedance spectra were analyzed using the ZView 4 fitting procedure (Scribner Associates Inc., Southern Pines, NC, USA). The results are presented as the logarithm of the impedance modulus as a function of the frequency. The value of the logarithm of the impedance modulus at a frequency of 0.1 Hz allows us to determine the protective properties of the paint system. Table 3 shows the EIS measurement sites.

#### 2.1.3. Assessment of Coating Degradation Using FTIR

Topcoat samples were collected from the tested bridges in order to determine the degree of degradation of the coating by means of infrared spectroscopy (FTIR). The samples of the topcoats were selectively collected from objects (by scraping), protected from external conditions and characterized using a Nicolet iS 10 Thermo Scientific FTIR spectrometer (Thermo Fisher Scientific Inc., Waltham, MA, USA) in the wave number range of 4000–600 cm^−1^. The spectra of paint coatings were acquired using the internal reflection-based method (ATR) (Attenuated Total Reflectance) at a resolution of 4 cm^−1^. The crystal used was diamond. The expanded uncertainty of peak determination (for 95% confidence level and expansion factor k = 2) is 1.4 cm^−1^. The research was conducted in comparison with samples prepared in the laboratory, made of the same paints as the ones used on the bridges. The results of the tests are presented in Section 3 in comparison with the results of the laboratory tests.

### 2.2. Accelerated Corrosion and Aging Testing of Coatings on Test Panels and Evaluation of Coating Damage

In the most frequently used procedure for the assessment of anti-corrosion systems, selected tests of accelerated tests are carried out in the laboratory [4,5,15,16,17,18,19,20,21,22,23,24,25,26,27], and tests are carried out simultaneously in natural exposures at corrosive stations. This is the most reliable assessment, but it requires a long period of research—even several years. The project used “reverse” research. Corrosion damage in natural operating conditions was assessed, where the corrosion exposures corresponded to C4/C5 exposures according to ISO 12944-2:1998; then, selected accelerated tests in the laboratory were performed, and the time after which the damage was similar to the damage on bridge structures was assessed. This allows for the selection of the most appropriate tests in a short time. Two tests were selected: the most widely used neutral salt spray test and the most popular in Europe variable exposure test according to ISO 20340: 2009 [28] (also introduced to the last edition of the basic standard for coating protection of steel structures ISO 12944-9: 2018 [3]).

According to ISO 12944-9: 2018 [3], the research in variable cycles consists of 25 cycles; each cycle lasts 7 days and includes the following: 72 h of exposure in a UV chamber (4 h UV, 60 °C, UVA lamps 340/4 h condensation, 50 °C) 72 h of exposure in a salt chamber (salt spray, 35 °C); 24 h in low temperature conditions (−20 °C). The cycle is repeated 25 times (total 4200 h). Passing this test is to ensure a shelf life of H (over 15 years in the C5M environment). The same requirements are set by NORSOK M-501: 2012 [29].

The salt spray test in accordance with ISO 9227: 2012 [30] was carried out for 4320 h to compare damage after a similar exposure period, although ISO 12944-6:1998 only requires 1440 h for the H durability period (a salt spray test is also required). One plate without incisions and two plates incised for each system were tested. Three T-shaped samples were also tested. Before and after the corrosion tests, the adhesion of the coatings was determined using the peel-off method in accordance with ISO 4624: 2002 [31]. Damage was assessed according to ISO 4628-1 ÷ 5, 8: 2003 [8] and ISO 4628-6:2011 [8].

### 2.3. Assessment of Coating Degradation Using FTIR an EIS

The barrier properties of the coatings were assessed, as in the field tests, by electrochemical impedance spectroscopy (EIS) according to ISO 16773-3: 2009 [32]. Laboratory tests were carried out in a three-electrode system, where the working electrode was the tested sample, the counter electrode was a platinum mesh, and the reference electrode was the calomel electrode.

The degree of degradation of coatings after 1000 h of exposure of samples to UV radiation in the QUV chamber, Q-Panel, according to ISO 16474-3: 2013 [33], was also tested. UVB 313 lamps were used, and the cycle was: 4 h UV/60 °C + 4 h condensation/40 °C. After exposure, the degree of chalking of the topcoats was assessed in accordance with ISO 4628-7: 2003 [8], as well as the change in their structure by infrared spectroscopy (FTIR) using the Nicolet iS10 spectrophotometer (Thermo Fisher Scientific Inc., Waltham, USA), and the change in the surface profile using the MicroProf (FRT GmbH, Bergisch Gladbach, Germany) non-contact instrument. The parameters of the surface geometry measurements are: measurement area 10 × 3 mm, resolution 1200 × 1000 points, acquisition and processing of measurement data-MARK III program. Measuring head parameters: CWL optical chromatic head type (Chromatic-White-Light Sensor), vertical resolution (z axis) 30 nm; 5 µm xy resolution with single pass, multiple passes of the head increase the resolution. The scanning area in the xy-axes was 10 × 10 mm and in the z-axis 3 mm. From this area, representative fragments of measurement fields were selected for illustration.

## 3. Results

The results of the assessment of coatings on objects according to ISO 4628: 2003, ISO 16276-2: 2007 and ISO 19840: 2012 are given in Table 4.

Table 5 shows the results of measurements on individual bridge structures and the average values of these measurements.

**Figure 2:**
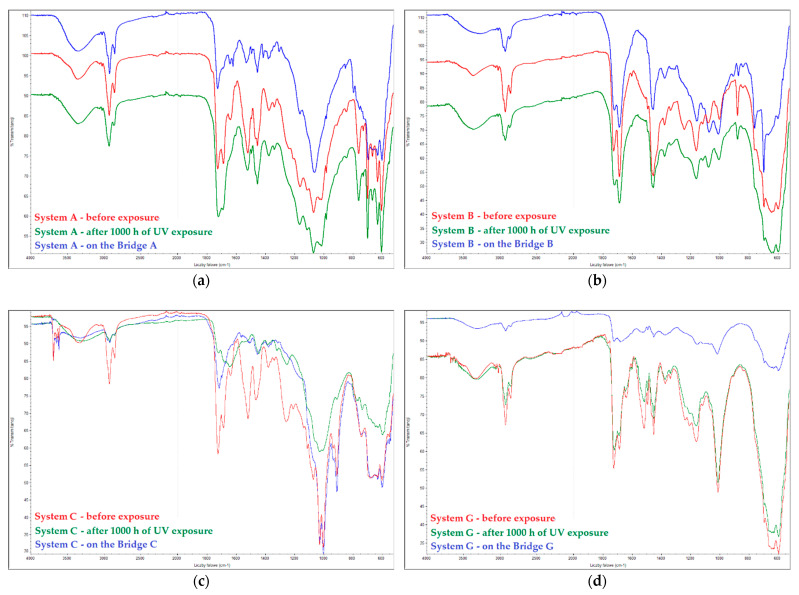
Spectra of topcoats in coating systems: (**a**) system A; (**b**) system B; (**c**) system C; (**d**) system G. Red line, before exposure; green line, after 1000 h of exposure in the UV chamber; blue line, on bridges.

**Figure 3:**
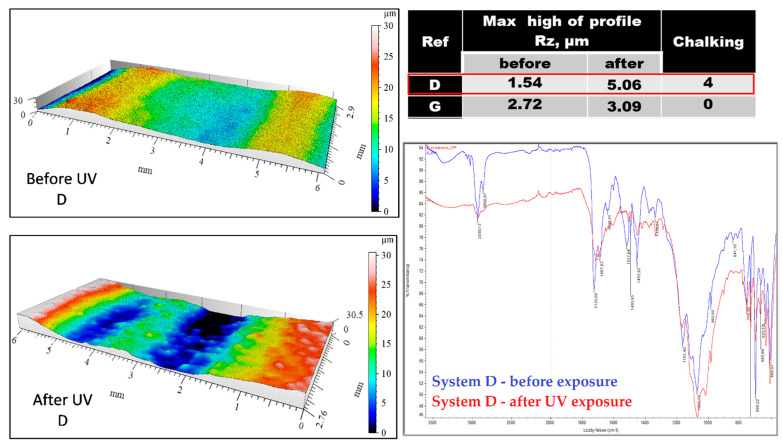
Surface profile and structure of the coating D before and after exposure to UV: greater change in roughness → changes in the FTIR spectrum.

The results of the tests on the degradation in the form of chalking of coatings made in the laboratory and the ones removed from bridges are presented in Table 6 and Figure 2 and Figure 3. The degree of chalking determined for coatings applied on bridges does not always coincide with the degree defined in the laboratory for the respective systems. The degree of chalking in some cases is greater in natural conditions than it is in the test chamber (Table 6). After over a dozen years of service, the structure of coatings on bridges looks similar to the structure of coatings exposed during 1000 h in a UV chamber. The bands generated by coatings exposed to natural atmospheric conditions on bridges remain the same or disappear in the same way as in the laboratory conditions (Figure 2).

**Figure 4:**
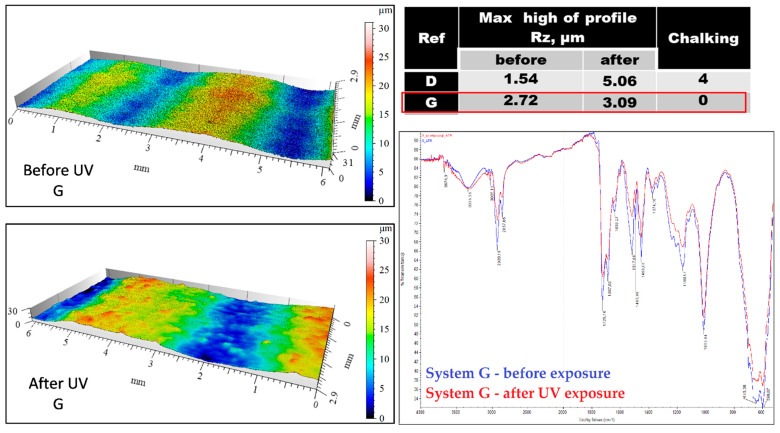
Surface profile and structure of the coating G before and after exposure to UV: smaller change in roughness → identical spectrum with spectrum before exposure.

The roughness of the coatings after exposure varies to a different degree, depending on the coating system, in agreement with both chalking and changes in the binder structure (Figure 3 and Figure 4). A large change in the surface profile of coating D corresponds to a high degree of binder degradation (Figure 3), while the lack of changes in the structure of the coating G agrees with an insignificant change in the surface profile (Figure 4).

**Figure 5:**
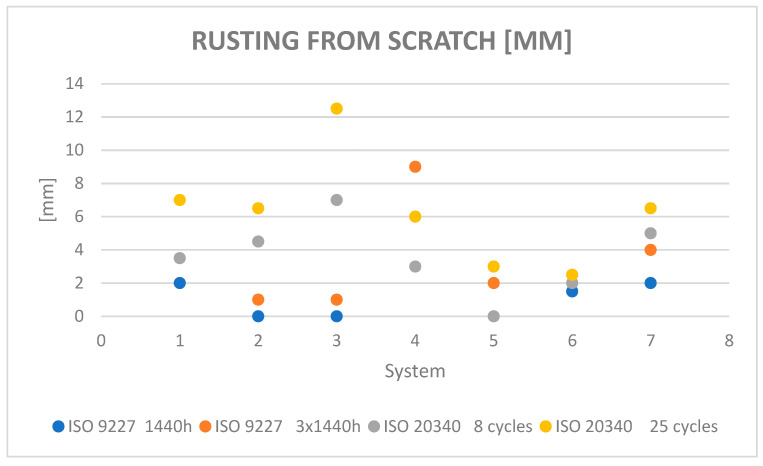
Rusting from the scratch measured according to ISO 4628-8:2012 [8].

**Figure 6:**
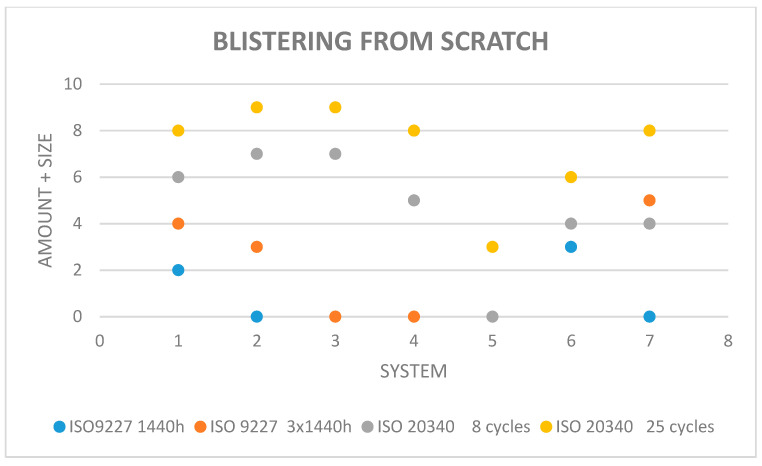
Blistering from the scratch measured according to ISO 4628-8:2012 [8].

The results of corrosion and blistering of the coatings around the crack according to ISO 4628-8:2012 [8] are given in Figure 5 and Figure 6. Other damages during exposure in the salt chamber were not found.

**Figure 7:**
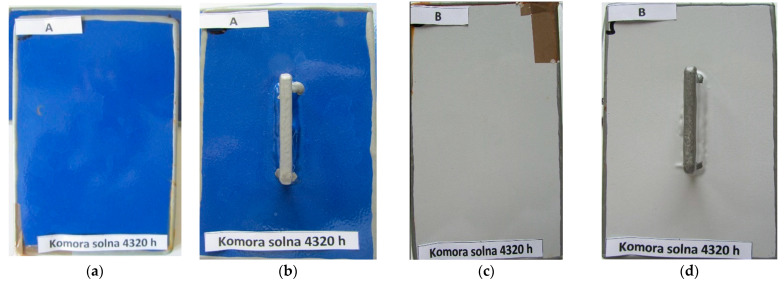
The appearance coatings without scratches after 4320 h of exposure in a salt spray chamber: (**a**,**b**) system A; (**c**,**d**) system B.

**Figure 8:**
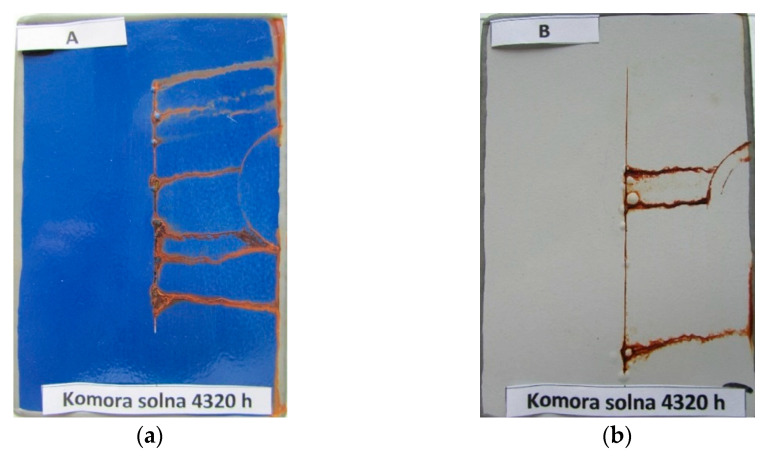
The appearance of coatings with scratches after 4320 h of exposure in a salt spray chamber: (**a**) system A; (**b**) system B.

Figure 7 shows an example of the appearance of the coatings without the scratch after 4320 h of salt spray testing. The same is true for the other systems and tests, where no differences were found between the results for flat and T-shaped plates. The appearance of A and B system coatings with scratches after 4320 h of exposure in a salt spray chamber are presented in Figure 8.

**Figure 9:**
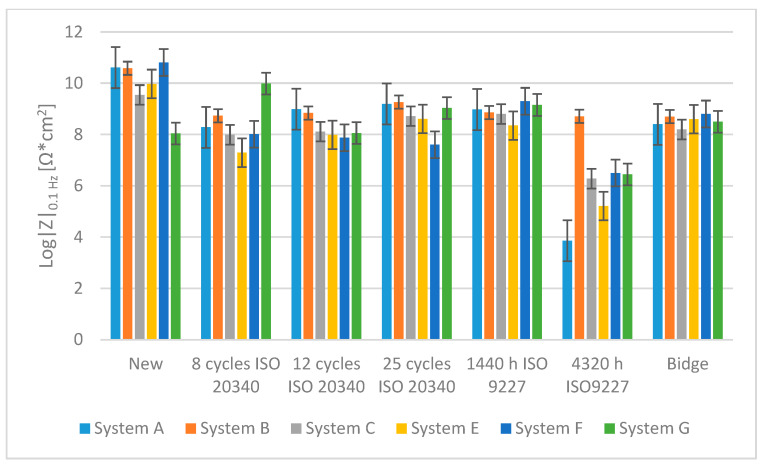
Values of log|Z|0.1 Hz before accelerated tests, after accelerated tests and on the bridges.

Figure 9 summarizes the measurement results of log|Z| at 0.1 Hz for new systems, for systems after various accelerated tests and for systems on the bridges.

Table 7 shows the adhesion values of the new systems in comparison with the adhesion of the systems after the salt spray tests and after 25 cycles according to ISO 20340:2009 [28].

## 4. Discussion

The corrosion resistance of all systems under real conditions on site after the tested period of operation (10–17 years) is very good, both when visually assessed according to ISO 4628: 2003 [8] and assessed by electrochemical impedance spectroscopy. The assessment by the EIS method shows slight differences between the results obtained at different measurement sites, but out of 197 measurements, only one result of logarithm of the impedance modulus is below the critical value for barrier properties of 6 (equal to 5.9), two results are below the value of 7, and most of them are between 8 and 10 and are 1–2 orders of magnitude lower than the baseline values obtained in the laboratory on the same systems.

The EIS method cannot be used to unambiguously determine the most endangered areas on bridge structures.

Local damage can be seen in areas of underpainting or significantly lower thickness of the systems than the specified thickness. Local damage also depends on the structure of the bridge: the number of gaps, sheet packets or nodes. These places require special care both in the design and during application and supervision. Of course, the old lattice bridges have more such places and there are more local damages on them, but they are not related to the type of paint (it is known from other authors’ experiences that in such places the old generation paints (red lead or Chromium (VI) pigmented ones) with a very high degree of penetration are the most effective. 

The systems are also characterized by good adhesion to the substrate and maintaining adhesion during exposure to corrosive agents. After accelerated tests, the adhesion did not change significantly in relation to the initial adhesion (Table 7). Clear differences between the coatings can be seen in the degree of chalking.

Looking at the results of accelerated tests in the salt spray chamber and in variable cycles, it can be seen that no corrosion changes occurred on the tiles without scratch in the analyzed period. There were also no differences in the results of corrosion tests between flat and T-shaped plates, as illustrated, for example, in Figure 6.

Different failures appear around the scratches, as shown in the example of coating systems A and B (Figure 7). In any case, the damage is greater for the tests in alternating cycles than for the tests in the salt spray chamber, even over extended periods of time.

Blisters around the scratch appear as follows: with system A on all tests; for B in alternating cycles and long salt spray test; for C and D only in alternating cycles; for E only in the long test in variable cycles; for F in all tests; for G in alternating cycles and in long salt spray tests.

Crack corrosion occurs as follows: for system A in all tests; for systems B and C only in alternating cycles; for system D in all tests; for system E in all tests, but minimal; for system F in all tests, for system G in the long salt spray test and in the variable tests.

There is no better performance of coatings with any special pigment, neither the sacrificing ones such as zinc, nor chemically protecting such as aluminum and zinc phosphate, and not even in those ones increasing the barrier properties of coatings such as iron flakes.

In the EIS tests, after accelerated tests in variable cycles (after 8, 12 and 25 cycles) and after 1440 h in the salt chamber, a decrease in the value of log|Z| at 0.1 Hz by 1–2 orders of magnitude is visible for all systems. There are no clear differences in the results for the coatings after 8, 12 or 25 cycles. A certain increase in the results for 25 cycles may be associated with the formation of corrosion products already under the coatings, temporarily sealing the system. A different situation can be seen for most systems (except for system B) after 4320 h of salt spray testing. There was a significant decrease in log|Z| at 0.1 Hz to 4–6. This probably proves that soluble corrosion products were washed out and that the barrier properties of the system were significantly reduced.

The damage around the crack does not correlate with the behavior of the coatings on the bridge structures during the tested period of operation. An interesting question concerns whether there will be any connection between this damage and the behavior of coatings on bridges over a much longer period of operation, or in areas of mechanical damage (natural or deliberately carried out as part of planned research).

Knowing the recipes of the paints, one could possibly link the degree of damage to the protective mechanisms of each system, but that was not the task of this project.

Topcoats (chalking resistance) show the greatest differences between the systems. Tests in simulated atmospheric conditions, in a UV chamber, revealed a similar mechanism of degradation as in coating systems exposed to natural conditions. FTIR spectra of the coatings removed from bridges and of laboratory samples show that bands characteristic for polyurethane binders either disappear or remain in place. Certain differences occurred in terms of the intensification of the chalking phenomenon. Chalking of coatings on objects in all cases, except for system A, for which no chalking was found both in the laboratory and on the object, is higher on site than in laboratory tests. The damage mechanism, inferring from the FTIR spectra, is the same in both cases. This proves that the time of laboratory tests is too short, according to the EN 13523-10: 2010 standard [34]. It seems that the recommendations contained in the requirements of the GSB ST 663-4 standard for powder coatings [35], requiring the Florida test, allow for a much better determination of this property of coatings. However, it is connected with a longer and more expensive test cycle.

## 5. Conclusions

The above discussed results show that the standard protection with an epoxy/polyurethane system with thickness as recommended in ISO 12944-5: 2009, without special active fillers, is able to ensure high durability in a C4/C5 environment because on all tested bridges, the paint systems retained their anti-corrosion properties after 10–17 years of operation.

The greatest differences in the durability of anti-corrosion systems result from the design of the protection (protection of gaps) and the quality of workmanship (presence or absence of supervision and its quality).

The results of the laboratory tests conducted indicate the following: 

(a) ISO 9227: 2012 test is not suitable to assess the behavior of the systems;

(b) The shortening of the ISO 20340: 2009 test to the 14 cycles is possible because the same EIS value as on the bridges appears between 4 and 14 cycles and showed no meaningful change up to 25 cycles, which shows that the ISO 20340: 2009 test is too severe with a requirement of 15 years durability in the C5 environment;

(c) Duration of laboratory tests in the UV chamber should be extended because chalking of coatings on objects is higher on site than in laboratory tests;

(d) Variable corrosive conditions produced both heavier blistering and rusting around scratches than constant ones did in salt chamber and during the laboratory tests damages (blistering, rusting) occurred only on scratched coatings.

Meeting environmental regulations and reducing production costs remain a key challenge and a major driving force for new developments in anticorrosive coatings, however clear relationship between protective properties and pigmentation of coating systems could not be proved during the study. These results should not discourage research into new material solutions, in view of the 100 years of corrosion protection as a possible target, as it appears in many long-term service life projects planned by investors.

## Figures and Tables

**Table 1:** Objects with the applied coating systems and the characteristics of operating conditions.

Bridge Number/ Coating System According to Table 2	System Service Life [Years]	Corrosivity Category ^1^	Structure Appearance (Characteristic Photo)
Kośmin Bridge 1/A	13	C4	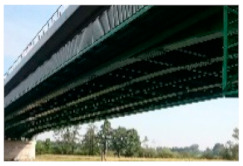
Tryńcza Bridge 2/B	10	C4	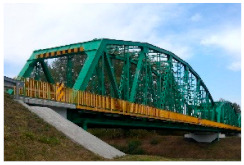
Góra Kalwaria Bridge 3/C1	16	C5I	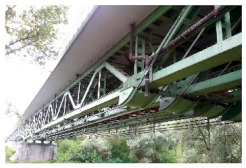
Gdański Bridge in Warsaw 4/C2	17	C5I	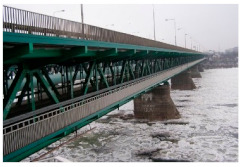
Kazimierza Wielkiego Bridge 6/E	16	C4	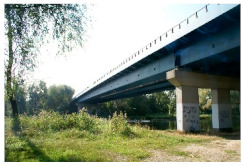
Fordon Bridge in Bydgoszcz 7/F	15	C5I	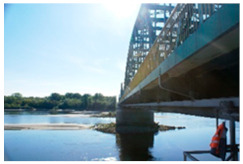
Praski Bridge in Warsaw 8/G	15	C4	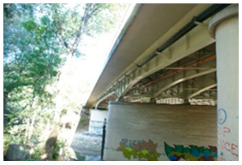

^1^ Corrosivity categories are given in accordance with the edition of ISO 12944-2: 1998.

**Table 2:** Types of applied coating systems.

System	Coating Type	Resin/Curing Agent/Anticorrosive Pigment
A	Primer	EP (HS)/amine adduct/Al (2–4 wt.%)
	Intermediate	EP (HS)/polyamine/Al
	Topcoat	PUR (acrylic)/HDI
B	Primer	EP (HS)/polyaminoamide/Al (2 wt.%)
	Intermediate	EP (HS)/polyaminoamide/Al (2 wt.%)
	Topcoat	PUR (acrylic)/HDI
C	Primer	EP/polyamide/Zn (75 wt.% in a dry coating)
	Intermediate	EP/polyamide/Al (1–2.5 wt.%)
	Topcoat	PUR (acrylic/polyester)/HDI
D ^1^	Primer	EP (HB)/polyamine/ion exchange pigment
	Intermediate	EP (HB)/polyamine/–
	Topcoat	PUR (acrylic)/HDI
E	Primer	EP/polyamidoamine/Zn (94 wt.% in a dry coating)
	Intermediate	EP/polyaminoamide/MIOX (58 wt.%)
	Topcoat	PUR (acrylic)/HDI/MIOX (47 wt.%)
F	Primer	EP/polyaminoamide/Al (10 wt.%)
	Intermediate	EP/polyamine/MIOX (12 wt.%), Al (10 wt.%), Zn phosphate (5 wt.%)
	Topcoat	PUR (acrylic)/HDI
G	Primer	EP/polyaminoamide/Zn phosphate (10.6 wt.%)
	Intermediate	EP/polyaminoamide/MIOX (36.5 wt.%)
	Topcoat	PUR (acrylic)/HDI

^1^ D system was tested only in the laboratory.

**Table 3:** EIS measurement sites on bridge structures.

Bridge/ Coating System According to Table 2	EIS Measurement Sites
Kośmin Bridge 1/A	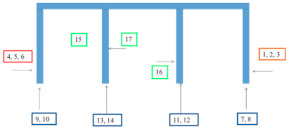
Tryńcza Bridge 2/B	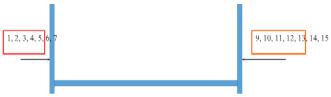
Góra Kalwaria Bridge 3/C1	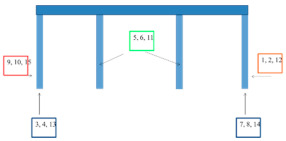
Gdański Bridge in Warsaw 4/C2	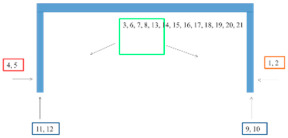
Kazimierza Wielkiego Bridge 6/E	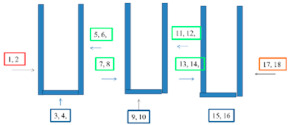
Fordon Bridge in Bydgoszcz 7/F	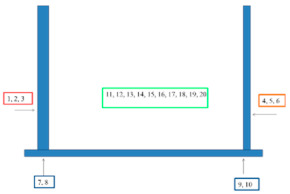
Praski Bridge in Warsaw 8/G	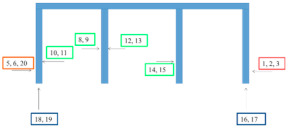

**Table 4:** The results of the assessment of coatings on objects according to ISO 4628: 2003, ISO 16276-2: 2007 and ISO 19840: 2009.

System	Average Thickness, µm	Adhesion Degree	Degradation Degree, General Comments
A	207 ± 11.8	0	no degradation
B	447 ± 14.5	0–2	chalking 2, rust degree Ri1, crevice corrosion
C1	252 ± 11.2	2	chalking 2
C2	410 ± 16.2	2–3	chalking 1, on the lower flange of the girder chalking 2, rust degree Ri1
E	281 ± 5.1	2	chalking 3
F	365 ± 8.0	0	chalking 1, crevice corrosion
G	188 ± 5.2	1	chalking 1, corrosion on sheet packages and on the surface of the lower girder flange

**Table 5:** Log|Z| at 0.1 Hz values at individual measurement sites and their average values.

Measurement Site	System						
	A	B	C1	C2	E	F	G
	Log|Z| at 0.1 Hz Values						
1	9.1	8.6	8.1	6.6	9.4	9.6	7.7
2	8.7	10.8	8.2	6.5	6.7	10.2	7.8
3	8.6	8.9	8.7	9.7	8.8	9.4	8.1
4	9.0	7.5	6.0	6.4	8.6	8.3	7.1
5	8.8	9.2	5.7	7.2	9.7	8.6	9.7
6	8.5	10.0	8.3	6.7	9.6	5.6	9.4
7	8.3	6.5	9.0	9.9	7.6	9.1	10.3
8	8.3	8.5	8.0	9.7	8.8	9.0	6.5
9	8.3	8.6	8.4	8.9	7.6	10.1	8.8
10	8.9	8.4	8.4	7.7	7.4	9.3	9.7
11	7.6	8.9	8.4	7.0	8.0	7.4	6.7
12	8.6	8.8	8.9	6.9	9.8	7.5	9.8
13	8.6	9.0	8.9	7.3	9.9	7.9	6.2
14	7.5	7.9	9.0	10.9	8.2	9.9	9.8
15	8.0	8.8	8.6	6.7	7.8	9.8	8.5
16	8.2	8.8	-	10.7	8.3	9.0	9.0
17	8.4	-	-	10.5	9.6	9.2	9.2
18	-	-	-	10.4	9.6	10.5	8.0
19	-	-	-	7.5	9.3	10.5	7.9
20	-	-	-	8.5	-	7.8	12.5
Average	8.4 ± 0.2	8.7 ± 0.5	8.2 ± 0.5	8.2 ± 0.7	8.6 ± 0.4	8.8 ± 0.6	8.5 ± 0.7

**Table 6:** Comparison of chalking degree between the coatings after 1000 h exposure in a UV chamber and coatings on bridge structures.

System	Chalking Degree after UV Chamber	Chalking Degree on the Bridge
A	0	0
B	0	2
C	1	1–2
D	4	–
E	0	3
F	0	1
G	0	1

**Table 7:** Coating adhesion before and after salt spray tests according to ISO 16276-2:2007 [28].

System	Average Adhesion of the Coatings without Scratches, MPa		
	New	After Salt Chamber Test	After 25 Cycles
A	12.5	10.0	8.8
B	12.1	11.0	12.1
C	7.8	7.5	6.2
D	8.1	8.2	8.0
E	4.2	5.2	5.1
F	8.2	9.1	8.1
G	12.5	10.0	8.8

## Data Availability

Not applicable.
